# Peer Review of “Supporting Technologies for COVID-19 Prevention: Systemized Review”

**DOI:** 10.2196/38606

**Published:** 2022-05-24

**Authors:** Mathew Mbwogge

**Keywords:** COVID-19, medical treatments, personal protective equipment, testing methods

*This is a peer-review report submitted for the paper "Supporting Technologies for COVID-19 Prevention: Systemized Review"*

## Round 1 Review

### General Comments

The need for effective and rapid response mechanisms to the COVID-19 pandemic has seen the emergence of new technologies. The European Parliament has organized such technologies into 10 broad categories. Many studies have reported the emergence of new digital tools as a direct response to COVID-19. While some of the studies report that these technologies make a major impact on the management of COVID-19 despite some challenges in their real-life usage, others acknowledge that COVID-19 control is critical, which calls for regular stocktaking, given the rapid advances in the field. Following the above, the authors of the paper “Supporting Technologies for COVID-19 Prevention: Systemized Review,” [1] in an attempt to stay on top of these advances, investigated the emerging technologies relating to the COVID-19 pandemic. The topic addressed in this paper is of interest to the journal’s readership and the international community. Being an important topic, it would have been important to report the review based on specific reporting guidelines to make it more appealing. The paper does not comply with the journal guidelines. Apart from the lack of a research objective, the paper is lacking in its methodology due to the lack of use of reporting guidelines. As such, the results remain doubtful. The general structure and English warrant improvement. If this paper must be brought to standard, the following specific comments are worth considering.

### Specific Comments

1. The title of the paper does not conform to the journal guidelines.

2. The abstract of your paper needs to be structured following the recommended guidelines.

3. This paper neither has a research objective nor question to permit its evaluation.

4. You need to follow the guidelines of the journal to which you are submitting.

5. Kindly refer to the new PRISMA (Preferred Reporting Items for Systematic Reviews and Meta-analyses) checklist to see how you can report your search results.

6. You need to have a look at the reviews published in the journal you are submitting to.

7. The English of your paper needs to be improved.

8. The Methods section lacks clarity and warrants improvement.

9. Your references need to be in line with the journal guidelines.

The above specific comments are further divided into the below major and minor comments.

#### Major Comments

1. Firstly, you need to identify and report the type of review you conducted, to help in the evaluation of your paper. If this is a narrative review, kindly indicate clearly in your paper.

2. The title of your paper needs to be structured in line with the journal guidelines. I suggest the following: (1) Emerging Medical Technologies for Fighting COVID-19: Systematic Review; or (2) Emerging Medical Technologies for Fighting COVID-19: Narrative Review

3. Your abstract needs to be structured in line with the journal guidelines, to include the Background, Objective, Methods, Results, and Conclusion subsections. Additionally, be aware that the PRISMA checklist also provides additional information that must appear in the Abstract section of systematic reviews.

4. Kindly restructure the manuscript using the IMRD format using the following word template;

5. It is absolutely important to read through the journal guidelines to which you are submitting.

6. Kindly put your study in context as part of your introduction. Use the provided reference if you need help with how to put your study in context.

7. This study is without a research objective. State your research question and objectives.

8. Kindly report the Methods section using the subsections below:

- Study objectives
- Eligibility criteria for selected studies
- How literature was searched
- The method used to synthesize results
- Data management and analysis
- Quality assessment (including the risk of bias assessments)
- How missing data were handled
- Heterogeneity assessment
- The method used to present data and results

The above may vary depending on the type of review you undertook. A simple literature review of emerging technologies will normally not require some of the above subsections.

9. It is very important to indicate the guidelines used to report your review results.

10. Your results section should be reported based on your research objectives (yet to be defined) and should include the following:

a. Search results: [a] flow diagram based on the new PRISMA flow chart and [b] characteristics of included studies (table and discussion).

b. Risk of bias assessment

c. Synthesis results (report results based on objectives and the different technology categories)

d. Overall assessment of the body of evidence

e. Heterogeneity

Again, as highlighted above, a literature review will not require some of the above points (eg, assessment of overall evidence and heterogeneity). That said, if you carried out a narrative review, I suggest using the following reported guidelines. I also find the structure of this referenced narrative review and systematic review more robust (use these as references in reporting your review). In reporting a narrative review, it is important to bear in mind how narrative reviews are evaluated. Moreover, be aware that review papers are expected to be submitted with a filled template of the guidelines used.

11. You need to have a look at studies that have reported on similar topics for inspiration.

12. See guidelines for the structure of the Discussion section. Present your Discussion into (1) principal findings and (2) comparison with prior studies.

13. Kindly include a subsection “study limitations” as part of the Discussion section.

14. Your references have to be in line with the recommended journal guidelines. Set your reference manager to the American Medical Association (AMA) citation style and make sure to include a PubMed ID at the end of each reference. You can search the PubMed IDs of various articles at https://pubmed.ncbi.nlm.nih.gov. In the absence of a PubMed ID, kindly include a DOI (verify your DOIs using https://www.doi.org/).

15. Include a subsection “Author Contribution” after the Acknowledgments section to state the contribution of each author included in this paper.

16. Include a subsection “Conflicts of Interest” after Author contributions to declare any conflict of interest.

17. Kindly list all Multimedia Appendices before the References.

18. For referenced websites, ensure to make as much effort as possible to get and reference the PDF version of the article (ie, in the absence of a PMID and DOI).

19. Create a section “Abbreviations” after your references to list and expand all abbreviations in the text.

20. I suggest starting your Conclusion with a statement on the study objectives followed by a summary of findings, then lessons learned from your findings, and finally, suggested direction of future research.

#### Minor Comments

1. Kindly include only the corresponding author in the manuscript and create/include all coauthors in the metadata section of the online manuscript management system (MMS) of your journal profile.

2. End your introduction with the aim of the study.

3. Kindly format your table following the journal guidelines.

4. You may want to start your Table 1 with study ID, by merging columns 1, 2, and the last as 1 column. For instance, the first cell will be “Rendeki et al,” followed by “setting or country” in the second column, and then the description, etc.

5. Following from (24) above, I recommend having (1) a table of characteristics of included studies for each category of technology or (2) present a single table of “Characteristics of included studies” under the Search Results subsection of the Results section, after the PRISMA flow diagram.

6. I suggest attempting to format your Figure 1 following the new PRISMA diagram.

7. Review all your figures and their captions in line with the guidelines. Apart from being uploaded as Multimedia Appendices, all figures must appear in the body of the text where they are first mentioned. Use a single sentence as the caption for each figure, which should appear at the bottom of the figure.

8. Following from (7) above, you may want to combine figures (a) to (i) to form a single figure as is the case with Figure 4.

9. I advise downloading Grammarly to assist you with the editing of your paper.

10. There is a need to justify your outcome prioritization. I suggest organizing your technology categories in line with the European Parliament categorization.

11. Ensure that titles and subtitles of your “Comparison with Prior studies” subsection of the Discussion are the same as the titles and subtitles of your Results section (Prevention, Diagnosis, Treatment, etc), and as suggested in (10) above.

## Round 2 Review

### General Comments

I acknowledge that the authors of the paper titled “Supporting Technologies for COVID-19 Prevention: Systemized Review” [1] have done well to improve on the overall structure and presentation of the paper, with a much better flow. Comments that were made in the previous round were based on the understanding that this was a standard “systematic review” type paper, but this is not the case. However, this paper still warrants some improvements. Kindly refer to the below major comments.

### Specific Comments

#### Major Comments

1. Systematic reviews require a predefined robust search strategy that is exhaustive, has an appraisal scheme for each type of study (both risk of bias and quality) with well-cited tools, has a clearly outlined method for synthesizing results, has a method of assessing all the evidence emanating from the literature, and most especially, has a clearly stated guideline used in reporting the review. Given that this review does not formally appraise the included studies for risk of bias and quality, neither does it have a clearly outlined method of synthesis, it will be appropriate to identify your study either as a (1) literature review, (2) systemized review, (3) narrative review, or simply (4) overview, none of which forcefully require a comprehensive search and formal appraisal of studies, and are not typically aimed at a narrative synthesis. It is enough to note here that even “systematic reviews with narrative synthesis” and “rapid reviews” that may omit some aspects of a standard systematic review follow specific citable guidelines in their methods and synthesis approach, to say the least.

2. It is absolutely important to bear in mind that reviews have their terminologies, as is the case with randomized controlled trials or other studies. You wrote “In this paper, 150 news articles and scientific reports on COVID-19–related innovations during 2020-2021 were firstly checked, screened, and shortlisted to form a pool of candidates yielding a total of 18 publications for review” and yet elsewhere you said, “After the initial candidates were selected, they were subjected to eliminating evaluations.” I do not think the term “candidate” can be used to refer to records retrieved in reviews. You may want to rephrase those and elsewhere (Introduction and Methods sections) in the body of your text and use “records” or “articles” instead.

3. Your “results” and “conclusions” subsections of the Abstract are not robust in a way that helps the reader understand what you found and what you learned or deduced from the findings and your recommendations. Kindly include a sentence or two each for personal protective equipment, testing methods, medical treatment, and other considerations in the “results” subsection.

4. Kindly include the following phrase in the “methods” subsection of your Abstract: “The keywords ‘COVID-19 technology,’ ‘COVID-19 invention,’ and ‘COVID-19 equipment’ were used in a Google search to generate related news articles and scientific reports.” Additionally, indicate when (exact date) the search was performed.

5. Regarding your PRISMA diagram, your numbers for records identified from other databases (websites) do not add up. You excluded 15 articles from the 30 you sought to retrieve, and it follows that you apparently excluded all 15 articles you assessed for eligibility, but you contradictorily still included the 15 articles in this review. Kindly verify and correct your PRISMA chart.

6. Your PRISMA diagram shows that you searched other websites other than Google; it will be absolutely helpful and more robust to indicate these websites under your “Search strategy.”

7. You did well to have included the PRISMA flow. Kindly substantiate your phrase “The selection of the article followed the guideline of PRISMA 2020” with a suitable reference.

8. Under “Testing methods” in your Results section, kindly also allude to “pooled” and “rapid testing (serology and antigen)” technologies as these are indispensable innovations to increasing the turnaround time and for timely detection. This updated Cochrane review as well as this list of 42 rapid testing technologies considered to be of acceptable performance by the UK government can help you identify suitable new technologies to add to this review. Regarding their pros and cons, it might be worthwhile to also look at the extent to which information provided by manufacturers is helpful for each technology considered if possible.

9. Coming to your Study Limitations, your phrase “Also, the paper only provides a quantitative comparison between the technologies” does not seem to be coherent with your synthesis approach. I think this should be a qualitative comparison since you made use of textual descriptions to draw similarities and dissimilarities between the data. Tabular presentations facilitate the narrative but do not make it quantitative. Kindly phrase and include the following in your Study Limitations as well:

a. The search strategy was not comprehensive as it was limited to 1 database (Google).

b. The fact that the protocol was not registered with PROSPERO (international prospective register of systematic reviews) might have affected the results in one way or the other.

c. Even though you unveiled some of the complexities regarding supporting technologies, a quantitative analysis would have also added value to the review results.

d. You did not do a formal appraisal of the included studies and the overall evidence from included studies. This must have affected your results.

10. Tables 1 through 3 make up 17 articles instead of 18 according to the number of retained articles. Kindly verify.

#### Minor Comments

1. Your “Conflicts of Interest” should follow the journal guidelines. Kindly use “None declared.”

## Round 3 Review

### General Comments

Unfortunately, I still have the 3 following concerns.

### Specific Comments

#### Major Comments

1. Recommendation #3: I am happy that the authors of this paper [1] improved on the results following the recommendation in point 3, but this recommendation was primarily referring to the Results and Conclusion subsections in the Abstract. The current wording in the Results of the Abstract should be moved to the Methods subsection of the Abstract. This means that you are yet to produce a summary of your findings (results) in the Abstract. Additionally, kindly increase the word count of the Conclusion subsection in the Abstract to reflect the main Conclusion of the paper.

2. The authors have also done well to have deployed the current PRISMA (Preferred Reporting Items for Systematic Review and Meta-Analysis) flowchart. However, your flow diagram shows that you included 5 articles from a previous version of this review indicating this paper is about updating a previous review, and I do not think it is the case. Except otherwise, kindly leave this box empty and move this number (n=5) to either “Records identified from Databases” or “Records identified from Websites.” My humble suggestion is that since you seem to have identified 200 records from Google search and ScienceDirect, under “Records identified from Databases (n=200),” kindly specify “Google=150” and “ScienceDirect=50” for readers to be clear about how many articles were retrieved from which database. Under “Records identified from Websites,” kindly put “n=5,” assuming that the 5 previously published reviews were identified from websites. If these were identified through Google search, ScienceDirect, or Cochrane, then kindly include under Records identified from Databases and leave “Records identified from Websites” empty.

3. You need to correct your statement “Three previous review papers were also included” as this seems to be 5 in the flow diagram.

## Abbreviations

PRISMA: Preferred Reporting Items for Systematic Reviews and Meta-analyses
PROSPERO: international prospective register of systematic reviews
